# A settingless fault detection approach for MVDC network

**DOI:** 10.1038/s41598-026-38187-2

**Published:** 2026-03-04

**Authors:** Amr Kassem, Hossam Sabra, A. A. Ali, K. M. Abdel-Latif

**Affiliations:** 1 Department of Electrical Machine and Power Engineering, Faculty of Engineering, Helwan University, Cairo, Egypt; 2 Ministry of Electricity and Energy, South Cairo Electricity Distribution Company, Cairo, Egypt

**Keywords:** Fault detection, MVDC distribution networks, Voltage source converter, Engineering, Electrical and electronic engineering

## Abstract

Medium Voltage Direct Current (MVDC) distribution networks present a promising solution for modern power distribution, yet fast and selective fault detection remains a significant challenge. Conventional protection methods often rely on predefined threshold settings and struggle to distinguish between internal and external faults—especially in the presence of high-impedance conditions or outfeed scenarios. This paper proposes a setting-less fault detection algorithm that determines fault locations by evaluating the rate of change in the difference between local and remote terminal currents. The approach eliminates threshold dependency, enhancing adaptability, sensitivity, and robustness. Advanced signal processing techniques are employed to suppress line capacitance discharge effects and minimize data exchange between terminals. To validate the scheme, extensive simulations are carried out on a multi-terminal MVDC system using PSCAD/EMTDC and MATLAB. The proposed method successfully achieves fault detection times as low as 0.25 ms, accurately detects faults with resistances up to 200 Ω, and maintains robust operation under 50 dB Gaussian noise, sudden load changes, and AC-side disturbances. The results confirm the scheme’s effectiveness and suitability for real-world MVDC protection applications.

**Supplementary Information:**

The online version contains supplementary material available at 10.1038/s41598-026-38187-2.

## Introduction

MVDC distribution system acts as an intermediary between LVDC and HVDC systems, as well as providing integration with renewable energy resources, power loads, and smart grids^1^. MVDC network has become feasible due to the continuous advancements in Voltage Source Converter (VSC) technology. MVDC systems have gained significant attention in recent years due to their advantages in improving power transmission efficiency, reducing losses, and integrating renewable energy sources. Therefore, ensuring the safe and reliable operation of MVDC systems is crucial, which demands effective protection algorithms for fault detection. MVDC grids can enhance systems with a high penetration of DC loads and power converters. The number of DC loads is increasing, with up to 80% of loads in commercial and residential areas being DC^2^.

The DC protection system is a significant challenge since DC fault currents have a very large magnitude and frequently reach their peak within a few moments after fault occurrence, in addition to the defenseless VSC against DC faults^3^. IGBTs can defend themselves via self-protection blocking during the occurrence of the short circuit, and then the VSC functions as an uncontrolled full-bridge rectifier following the blocking of the IGBTs. Consequently, VSC controllability can potentially be lost and the fault current passing through the freewheeling diodes causes damage^4^. Therefore, the whole protection system should operate within milliseconds after fault inception. Multi-Terminal Direct Current (MTDC) systems require the continuous operation of the converters without interruption during the DC fault event^5^. To do this, fast and selective DC line protection is required to achieve resilient DC grids.

The DC protection methods discussed in the literature are primarily grouped into two categories: single-ended (non-pilot protection techniques) and double-ended (pilot protection techniques)^6^.

Single-ended protection uses only local information, and no communication between other relays is required to detect the faults. Employing single-ended methods are restricted by the existence of the installed current limiting inductors at the two terminals of the HVDC transmission lines as a physical boundary. However, due to the complexity of this system, installing the current limiting inductors for DC distribution systems is quite complicated. Hence, applying single-ended protection to protect the DC lines is a preferable choice to protect HVDC transmission lines as a primary protection^7^. The authors in^8^ proposed a fast dc short-circuit fault detection method for HVDC grids using the voltage across the dc fault-current-limiting reactor and validated the technique by simulating different fault cases. The idea presented in^9^ proposed a local measurement-based protection system for multi-terminal HVDC systems using Rate of Change of Voltage (ROCOV) and Rate of Change of Current (ROCOC) algorithms for primary, backup, and busbar protections. The ROCOV algorithm provides fast, reliable, and accurate primary protection against low and high resistance fault conditions, while the ROCOC algorithm is implemented in case of the failure of the primary protection. Similarly, the work introduced in^10^ presents an ROCOC × ROCOV-based fault detection scheme for MVDC networks, which enhances sensitivity and selectivity by combining voltage and current transient characteristics. The protection criterion outlined in reference^11^ is designed to discern between different types of faults within the MVDC-MMC network by analyzing the voltage characteristics and their variations during fault conditions, specifically differentiating pole-to-pole faults from external and internal faults based on reactor voltage behavior. The faults within the DC network are identified in^12^ through the examination of transient current magnitudes, employing a mathematical representation of the fault current behavior. Based on analysis and evaluation, it can be inferred that the methodologies presented in^8^-^12^ rely on the inclusion of series inductors on both line segments. However, it should be noted that these techniques are not compatible with MVDC systems that do not incorporate such series inductors. Moreover, the configuration parameters of the protected line and series inductors are crucial factors in determining the protection parameters of the aforementioned methods. It is important to acknowledge that these predefined parameters may not be adequate or appropriate for varying networks, which can subsequently impact the reliability and dependability of the protection algorithm. The polarity and traveling wave (TW) time of the fault current are introduced in^13^ to detect DC faults and identify the fault location. Nevertheless, the limited length of the Medium Voltage Direct Current (MVDC) distribution system presents a challenge in capturing the traveling wave. Additionally, implementing such a scheme may involve complexities, significant expenses, false alarms, and vulnerability to noise. The authors in^14^ introduced a traveling-wave (TW)-based scheme for identifying various types of DC faults in MVDC microgrids. The proposed scheme focused on the TW’s polarity and characteristics of the wave shape rather than the arrival timing. However, the effectiveness of the proposed scheme relies on the shape of the traveling wave and can be subject to interference from noise within a specific range. In contrast, paper^15^ utilizes Singular Spectrum Analysis (SSA) to enhance traveling wave detection and localization, achieving high accuracy even under high-impedance faults and noisy conditions.

In a double-ended protection system (pilot protection), protection relays are installed at both ends of the transmission line. The deployment of protection relays on both ends of the line enables the protection algorithms to optimally utilize the information derived from both ends, thus allowing for accurate fault identification. The pilot protection schemes outperform the non-unit protection schemes in terms of dependability and sensitivity to high fault resistance^16^. Moreover, the viability of pilot protection schemes has risen as a result of the development of cutting-edge communication systems for the future power grid^17^.

The work introduced in^16^ proposed a frequency-based current differential protection scheme that addresses the issue of line capacitance discharge current, which could affect the performance of the differential protection. The scheme is founded on frequency-based current differential principles, enabling the precise identification of internal faults through the assessment of the ratio between low-frequency and high-frequency energy components within the differential current. The scheme presented^18^ in relies on the comparison of the current status matrix obtained from the analysis of both the positive and negative pole fault currents direction. The work introduced in^19^paper presented a high-speed differential protection scheme for DC distribution systems. The proposed scheme is based on the natural characteristics of the differential current measurements and a centralized processing device is suggested to compare current measurements. A transient current correlation-based protection algorithm for DC distribution networks is proposed in^20^. The proposed approach makes use of correlation analysis of transient currents at both line terminals to detect faults. Combining the acceleration protection principle with the capacitor discharge current second-order derivative after a fault occurs is analyzed in^24^. The characteristic of the high-frequency backward traveling wave is suggested in^25^ to identify the fault location in HVDC.

Recent works also explore adaptive and resilient protection strategies. For instance, reference^21^ proposes a communication-aided scheme using grounding line current variations for single-pole-to-earth fault detection in MVDC cables, achieving high accuracy under high-impedance faults and measurement noise. Additionally^22^, introduces a DC distance relay based on local measurements and auxiliary components for fast and selective fault detection in meshed MVDC microgrids. Furthermore^23^, presents an adaptive grid-resilient protection method using Level Order Tree Traversal (LOTT) and Bidirectional Dial’s algorithm for multi-fault scenarios in multi-DC microgrid systems, demonstrating rapid fault isolation under simultaneous and multi-fault conditions.

Unlike existing unit protection schemes that depend on predefined threshold values and rigid configuration parameters, the proposed protection approach introduces a setting-less fault detection mechanism tailored for multi-terminal MVDC networks. Traditional algorithms suffer from sensitivity issues due to their reliance on system-dependent thresholds, which are influenced by factors such as topology, line parameters, fault impedance, and operating conditions. These dependencies complicate implementation and may lead to false tripping or missed fault detection under dynamic scenarios.

In contrast, the novelty of the proposed work lies in the following contributions:

- The algorithm eliminates the need for a predefined threshold by calculating a nominal value based on the rate of change of the difference between local and remote terminal currents. This setting-less approach enhances adaptability across different network topologies without additional tuning.
- The scheme accurately detects internal faults with fault resistances up to 200 Ω, a condition where many conventional algorithms fail due to unchanged power flow.
- The method maintains high performance even in the presence of communication delays and 50 dB Gaussian noise, demonstrating its practical viability for real-world deployment.
- A consistent logic based on the sign of the computed nominal value enables simultaneous protection of lines and buses without complex configuration or separate algorithms.

The rest of the paper is arranged as follows: The structure of the investigated multi-terminal MVDC network as well as a brief of the unit protection implementation challenges in DC networks are detailed in Section "Multi-terminal MVDC network architecture and challenges associated with implementing unit protection in DC networks". The proposed setting-less unit protection scheme for MVDC networks is described in Section "Proposed setting-less MVDC protection scheme". Numerous simulation cases using the PSCAD program are implemented in Section "Simulation results and evaluation" to validate the suggested method. Finally, the conclusion is provided in Section "Conclusion".

## Multi-terminal MVDC network architecture and challenges associated with implementing unit protection in DC networks

In this section, we present the topology and configuration specifics of the examined multi-terminal MVDC network, along with an analysis of the unit protection schemes applicable to DC networks.

### Multi-terminal MVDC network structure under study

**Fig. 1:**
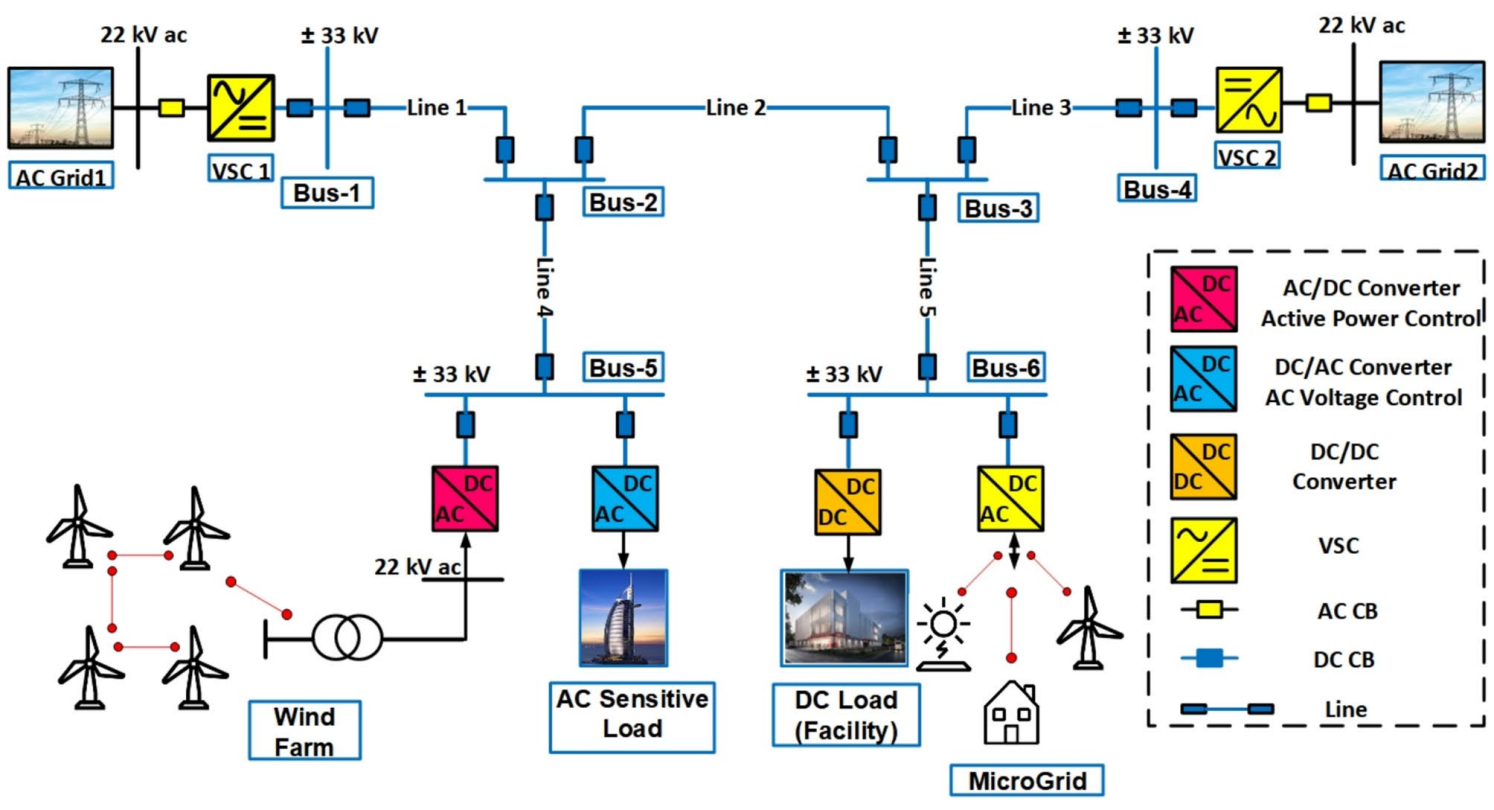
Two terminal MVDC networks.

For the MVDC distribution system, various topologies including radial, ring, and multi-terminal configurations are feasible. While the radial topology exhibits the lowest reliability, the ring topology is generally considered the most reliable albeit challenging to control and protect. In this paper, we focus on analyzing the protection mechanisms within a two-terminal MVDC system as depicted in Fig. 1, simulated using the PSCAD platform.

The MVDC network under study operates at a rated voltage of 33 kV and interfaces with AC networks through VSC1 and VSC2 at the two terminals. A unipolar symmetry structure is adopted, consistent with prevalent practices in MVDC distribution systems. Grounding of the DC system is achieved through the neutral point of the DC capacitor, providing a reference voltage for the entire distribution network. The system comprises five sections of DC cables connecting six DC buses. The DC buses (1 and 4) are interconnected with the AC grids (1 and 2) via 50 MVA VSCs (1 and 2) respectively. Bus (3) is linked to a DC load and a microgrid, while bus (5) is connected to an AC load and a wind farm. Detailed network parameters are provided in Table 1 for reference.

**Table 1:** The two terminal MVDC network data.

System voltages					
DC grid	33 kV				
AC grid	22 kV				
AC load	22 kV				
DC load	6.6 kV				
Converter station data					
Converter station	Power rating MVA		DC voltage kV	Operation mode	
VSC (1)	50		33	Q = 2 Mvar	Vdc = 1pu
VSC (2)	50		33	P = 30 MW	Q = 0
AC/DC of DG	50		33	P = 20 MW	Q = 0
DC/AC of AC load	50		33	Q = 2 Mvar	Vac = 1pu
DC/DC of DC load	15		6.6	P = 12 MW	Q = 0
Line data					
Line	Length (km)				
Cables 1, 2, 3	5				
Cable 4, 5	7.5				

### Unit protection implementation challenges in DC networks

The utilization of unit protection in DC systems has garnered significant attention due to its inherent advantages. Current differential protection techniques have been widely investigated in numerous studies for their applicability in DC distribution systems. The integration of the communication infrastructure in distribution systems, particularly with the advancement of smart grid technology, has greatly contributed to the widespread implementation of unit protection in DC distribution systems^24^. This section undertakes an analysis of the performance of unit protection schemes tailored for DC networks, aiming to evaluate their efficacy in facilitating rapid and precise fault detection within such systems.

The primary objective of this analysis is to address the complexities associated with implementing unit protection, ensuring that the desired performance metrics are achieved and quantifiable. Unit protection entails the establishment of a communication network to safeguard a designated zone. The seamless integration of communication infrastructure into distribution systems, particularly propelled by advancements in smart grid technology, has facilitated the widespread adoption of unit protection in DC distribution systems.

Unit protection offers distinct advantages, including high selectivity and immunity to several influencing factors such as the presence of distributed generation resources, fault resistance, the rate of change of fault currents, and fault current magnitudes. However, despite these advantages, several challenges arise during its implementation. One of the key challenges is the occurrence of capacitive transient currents caused by line discharges, which can inadvertently activate current differential protection during external fault conditions. Moreover, unit protection may face difficulties in detecting internal faults, particularly under specific outfeed conditions. Additional implementation challenges include issues related to timing, measurement synchronization, and determining precise threshold values, all of which significantly affect the overall performance of unit protection mechanisms.

To address the issue of distributed capacitance influencing current differential protection, various technical remedies have been explored, as outlined in reference^25^. These solutions involve compensating for capacitive currents and applying filtering techniques. In DC distribution networks, the effects of distributed capacitance on the line can be mitigated due to the relatively shorter length of distribution lines when compared to transmission lines. Consequently, the distributed capacitance is notably lower, particularly when compared to the parallel capacitance on the DC side. Nonetheless, reference^26^ advocates for the use of compensation techniques to further enhance system security by addressing this issue.

To address this challenge, reference^27^ indicates that the frequency of the distributed capacitive discharge current during a fault exceeds 1 kHz, as the traveling wave voltages during the fault cause charging and discharging of the distributed capacitance along the transmission line. In contrast, the frequency of the DC link capacitive discharge current falls below 1 kHz. By employing a wavelet transform tool, the low-frequency component of the measured current can be isolated by calculating the approximation coefficients. This approach effectively extracts the fault current while mitigating inaccuracies caused by the distributed capacitive discharge current.

Another significant challenge faced by differential protection systems in Medium Voltage Direct Current (MVDC) networks is communication latency. The speed at which differential protection operates is highly dependent on several factors, including measurement preparation, internal relay data processing delays, algorithm execution time, circuit breaker response times, and communication channel delays. Mitigating communication latency is essential to improving the performance of protection schemes. To address this challenge, it is necessary to adopt strategies that reduce communication delays, streamline measurement processing, and implement simplified protection algorithms. Reference^28^ highlights the potential of the IEC 61850 standardized communication protocol, which facilitates faster and more efficient data exchange between protection devices in MVDC networks. By reducing communication latency and improving reliability, IEC 61850 has the potential to significantly enhance the fault detection and isolation capabilities of line differential protection systems. The subsequent section on communication infrastructure will further elaborate on the implementation of the IEC 61850 protocol, recognized as an international standard for communication between intelligent electronic devices in electrical substations^29^.

The motivation for this research can be summarized as follows:

- Existing fault detection schemes depend on predefined threshold values, which can lead to detection inaccuracies under varying fault conditions.
- Line discharge currents may cause false tripping, compromising system stability and reliability.
- Data transmission delays and communication latency introduce potential protection delays, reducing the scheme’s effectiveness.
- High-impedance internal faults with outfeed often go undetected, posing a significant risk to network security.

## Proposed setting-less MVDC protection scheme

The main goal of this paper is to propose an effective yet simple DC protection scheme that has the ability to overcome the aforementioned challenges related to DC unit protection. The protection scheme utilizes commercial Intelligent Electronic Devices (IEDs) and smart grid communication infrastructure to meet the requirements of the MVDC protection system.

The proposed protection scheme is based on the rate of change of the difference between the local and remote currents. The proposed scheme consists of two parts, working in tandem: one for detecting line faults and the other for bus faults. The first part related to line fault detection requires that an IED be installed at each terminal of the protected lines and share information among the other IEDs. To convey signal data between line terminals, the IEC 61850 communication protocol over optical fiber is employed. The IEC 61850 communication protocol is used to align the shared data in time to avoid synchronization errors. The second part related to bus protection is carried out locally between each bus’s attached IEDs using the IEC 61850 horizontal communication protocol through the Ethernet protocol (IEEE 802.3)^30^.

### Measurements preparation

Each IED measures the currents of the positive and negative poles and calculates the line-mode current using the transformation matrix provided in Eq. (1):

1 $$\left[\begin{array}{c}{I}_{0}\\ {I}_{1}\end{array}\right]=\frac{1}{\sqrt{2}}\left[\begin{array}{cc}1& 1\\ 1& -1\end{array}\right]\left[\begin{array}{c}{I}_{P}\\ {I}_{N}\end{array}\right]$$

Here, I_0_ and I_1_ represent the ground-mode current and line-mode currents, respectively, whereas I_P_ and I_N_ denote the positive and negative pole currents, respectively. This transformation mitigates the impact of mutual coupling on transient current analysis in DC networks.

Each IED extracts the approximation coefficient at level 4 (A_4_) from the line-mode current and transmits it, along with a timestamp, to the IED located at the opposite terminal. Consequently, each IED obtains the level-4 approximation coefficient of the line-mode current for both its own terminal and the opposite terminal. The extraction process is carried out as follows.

**Fig. 2:**
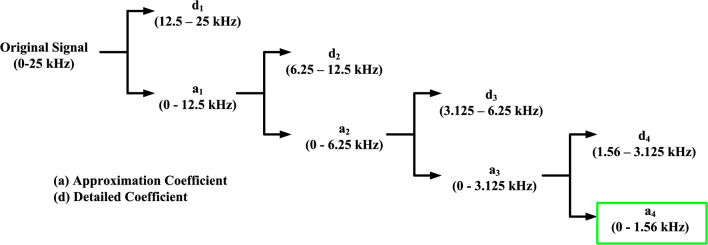
Decomposition of fault current using DWT.

According to IEC 61850, a sufficient sampling frequency for fault detection is approximately 50 kHz. Thus, this scheme employs a sampling frequency of 50 kHz. Based on Shannon’s theorem, the Nyquist frequency for this sampling rate is 25 kHz. To isolate the distributed discharge capacitive current by extracting the low-frequency component of the signal, a discrete wavelet transform (DWT) is applied up to level 4. The resulting approximation coefficient (A_4_​) encompasses the current within the range of 0 to 1.5625 kHz, as illustrated in Fig. 2.

It is important to note that the transformation matrix, which converts the positive and negative pole currents into line mode currents, followed by the extraction of approximation coefficients at level 4, significantly reduces the data dimensionality. This reduction in data volume facilitates efficient data transmission between the two ends of the line.

### Mathematical model for the proposed protection scheme

**Fig. 3:**
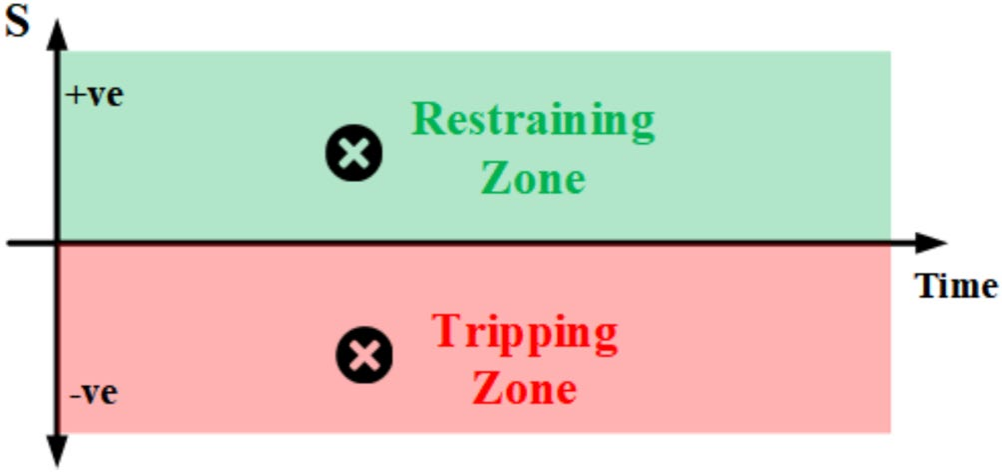
The graphical representation of the characteristic of the proposed scheme.

The characteristic of the proposed protection scheme is exemplified through an operational region and a restraint region, as depicted in the graphical representation shown in Fig. 3. The algorithm relies on the continuous monitoring of a computed nominal value (S), illustrated on the Y-axis with respect to time on the X-axis. The calculated nominal value S is a ratio between two magnitudes and is set to achieve the fundamental purpose of establishing the scheme’s independence from the threshold value that may influence the sensitivity and robustness of the protection scheme.

#### Model Input representation

- I_LOCAL_ represents the current measured at the local terminal.
- I_REMOTE_ represents the current measured at the remote terminal.

The difference in current between the two terminals is defined as:

$$X = I_{LOCAL} - I_{REMOTE}$$

$$Y = I_{REMOTE} - I_{LOCAL}$$

To analyze the rate of change of current, we compute the time derivatives:

$$\frac{d}{dt}X= \frac{d}{dt}({I}_{LOCAL}-{I}_{REMOTE})$$

$$\frac{d}{dt}Y= \frac{d}{dt}({I}_{REMOTE}-{I}_{LOCAL})$$

#### Definition of the operating index

The nominal value S is calculated based on the quotient of the rate of change of the difference between local current (I_LOCAL_) and remote current (I_REMOTE_) of the protected element. The S values is given by equation (2) as follows:

2 $$S = {\raise0.7ex\hbox{${\frac{d\left( X \right)}{{dt}} + \varepsilon }$} \!\mathord{\left/ {\vphantom {{\frac{d\left( X \right)}{{dt}} + \varepsilon } {\frac{d\left( Y \right)}{{dt}} + \varepsilon }}}\right.\kern-0pt} \!\lower0.7ex\hbox{${\frac{d\left( Y \right)}{{dt}} + \varepsilon }$}}$$

where, the coefficient Ɛ is added with a very small value to avoid dividing by zero.

#### Operational and restraint regions

- For restraint region: normal operation or external faults, both I_LOCAL_ and I_REMOTE_ have the same direction and magnitude. As a result, X = Y, the numerator and denominator are the same, and S equals 1.
- For Operational region: internal faults, I_LOCAL_ and I_REMOTE_ are in opposite directions and have different magnitudes. Thus, X ≠ Y and have opposing directions, and S yields a negative value.

### Line protection scheme

With respect to the line protection characteristic, the operating value S_L_ is calculated as the quotient derived from the rate of change in the difference between local current (I_LOCAL_) and remote current (I_REMOTE_) to the rate of change in the difference between remote current (I_REMOTE_) and local current (I_LOCAL_) of the protected line. The coefficient Ɛ is added with a very small value to avoid dividing by zero. The value of S_L_ for line protection is defined in Eq. (3) as follows:

3 $$S_{L} = {\raise0.7ex\hbox{${\frac{{d\left( {I_{LOCAL} - I_{REMOTE} } \right)}}{dt} + \varepsilon }$} \!\mathord{\left/ {\vphantom {{\frac{{d\left( {I_{LOCAL} - I_{REMOTE} } \right)}}{dt} + \varepsilon } {\frac{{d\left( {I_{REMOTE} - I_{LOCAL} } \right)}}{dt} + \varepsilon }}}\right.\kern-0pt} \!\lower0.7ex\hbox{${\frac{{d\left( {I_{REMOTE} - I_{LOCAL} } \right)}}{dt} + \varepsilon }$}}$$

### Bus protection scheme

For bus protection, the S_B_ is calculated as the ratio involving the rate of change of the total difference between the input currents entering the protected bus (I_IN_) and the currents output from that bus (I_OUT_) received from each IED attached to the protected bus to the rate of change of the total difference between the currents output from the bus (I_OUT_) and the bus input currents (I_IN_). Ɛ is a very small coefficient to avoid dividing by zero. Equation (4) defines S_B_ for bus protection as follows:

4 $$S_{B} = {\raise0.7ex\hbox{${\frac{{d\Sigma \left( {I_{IN} - I_{OUT} } \right)}}{dt} + \varepsilon }$} \!\mathord{\left/ {\vphantom {{\frac{{d\Sigma \left( {I_{IN} - I_{OUT} } \right)}}{dt} + \varepsilon } {\frac{{d\Sigma \left( {I_{OUT} - I_{IN} } \right)}}{dt} + \varepsilon }}}\right.\kern-0pt} \!\lower0.7ex\hbox{${\frac{{d\Sigma \left( {I_{OUT} - I_{IN} } \right)}}{dt} + \varepsilon }$}}$$

Each IED continuously monitors the operational state represented by point S on the characteristic plane, as depicted in Fig. 3. Under normal operational conditions and external faults, the operating point (S > 0) resides within the restraining region. This is because the currents at both terminals exhibit the same direction and rate of change.

In the event of an internal fault, the operating point (S < 0) enters the tripping region as the currents at both terminals exhibit opposing directions. For an internal fault with an outfeed case, the operating point (S < 0) remains within the tripping region, given the differing rates of change in currents between the two terminals.

### Communication infrastructure

**Fig. 4:**
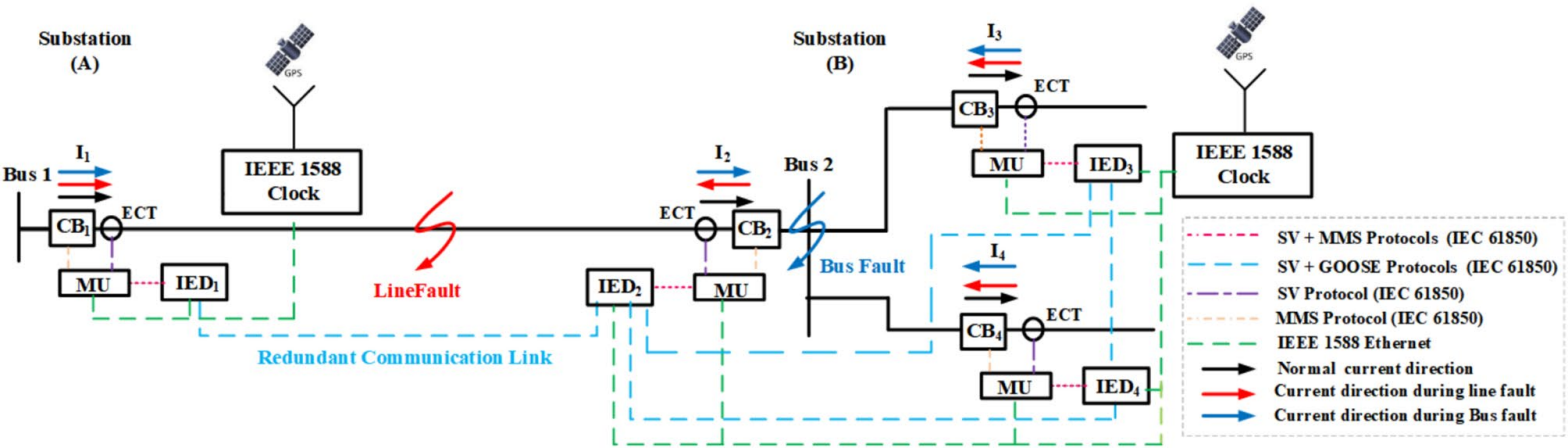
Communication infrastructure and operation methodology of the proposed protection scheme.

The communication infrastructure of the proposed scheme for line and bus fault protection is depicted in Fig. 4. IED_1_ and IED_2_ are responsible for detecting the line faults, while IED_2_, IED_3_, and IED_4_ are responsible for detecting faults on Bus 2. Each Electronic Current Transformer (ECT) is responsible for delivering the digital representation of the current signals to the Merging Unit (MU) over an Ethernet network using the Sampled Value (SV) protocol based on IEC 61850–9-2 and IEC 61869^31,32^. Then, the MU delivers the sampled value of the current signals to the associated IED, where the differential protection algorithm is executed. IEC 61850 is used to communicate the IEDs with each other through two redundant fiber optic channels to achieve line and bus differential protection to guarantee the system’s availability.

As line differential protection algorithms are sensitive to time synchronization, IEEE 1588 is used to provide precision.

time coordination between IEDs based on the precision time protocol (PTP)^33^. Each IED shares its decision parameter with the associated IEDs via the GOOSE messaging protocol based on IEC 61850. Finally, each IED compares the decisions and issues a trip command in the fault case to the associated circuit breaker via the Manufacturing Message Specification (MMS) protocol based on IEC 61850.

The typical causes of communication time delays are investigated to ensure that the protection system functions within the required timeframe. The various sources of time delay in the overall protection system are as follows:

- Current transducer processing time: The Hall-Effect current transducer has the capability of calculating the di/dt within a time delay of approximately 3 μs, given that it can handle up to 100A/μs^34^.
- Algorithm execution time: the proposed protection scheme is simple and does not require complex calculations. As a result, the microprocessor can handle the proposed protection algorithm in just a few microseconds.
- Communication time: communication delay is affected by the communication link’s speed and type, transmitted distance, and size of transmitted data. The communication delay is the time taken to transfer data bits from one node to another and is equal to the distance/propagation speed. The fiber optic propagation speed is typically around 180,000 to 200,000 km/s, resulting in a time delay of 5 to 5.5 μs/km^35^. For the case study mentioned in this paper, the farthest node considered is about 10 km away. Therefore, the communication time for sending and receiving the trip command will be about 100 μs. For fiber optic medium, the bandwidth can reach up to 500 MHz/km, and the size of the transmitted message only contains the current measurement sampled value. Thus, transmission delays can be neglected.
- Circuit breaker operation time: the operating time of the solid-state circuit breaker is less than 100 µs.

After accounting for all of the previously mentioned factors, the total delay time is expected to be around 250 s, which meets the time requirements of the protection system. As a result, the proposed protection scheme can be implemented using the proposed communication infrastructure capabilities.

### Operating principle of the protection algorithm

**Fig. 5:**
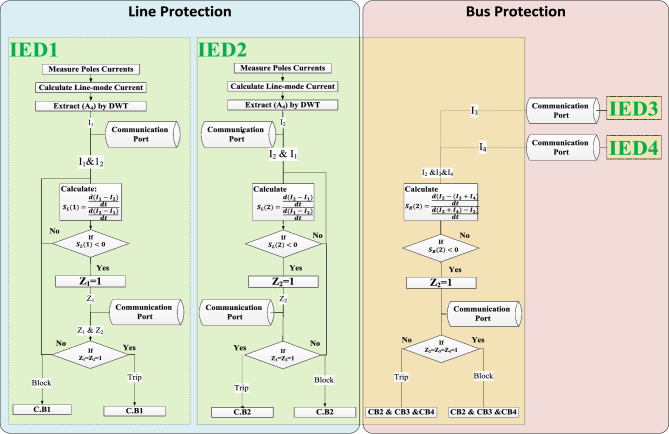
Flowchart of the proposed setting-less fault detection scheme based on real-time S-index evaluation, using IEC 61850-enabled IED interoperability.

The flow chart illustrating the proposed scheme is shown in Fig. 5. The procedure is explained as follows:

#### Operating principle of the line protection algorithm

1. Both IEDs attached at each terminal of the protected line communicate together and transmit the 4^th^ approximation coefficient of the line mode current values through the IEC 61850 communication protocol via optical fibers. Accordingly, each IED has local and remote currents.
2. Each IED calculates ${S}_{L}\left(\mathrm{i}\right)$ according to Eq. (5):
  5 $$S_{L} \left( i \right) = {\raise0.7ex\hbox{${\frac{{d\left( {I_{LOCAL} - I_{REMOTE} + \varepsilon } \right)}}{dt}}$} \!\mathord{\left/ {\vphantom {{\frac{{d\left( {I_{LOCAL} - I_{REMOTE} + \varepsilon } \right)}}{dt}} {\frac{{d\left( {I_{REMOTE} - I_{LOCAL} + \varepsilon } \right)}}{dt}}}}\right.\kern-0pt} \!\lower0.7ex\hbox{${\frac{{d\left( {I_{REMOTE} - I_{LOCAL} + \varepsilon } \right)}}{dt}}$}}$$

Where SL is the calculated value for the protected line as defined in equation (5-2), while i denotes the IED’s number and the value of $\varepsilon$ is a very small coefficient suggested by 1% of the rated line current to avoid dividing by zero.

Each IED continuously monitors its operational point S_L_; if the operating point of the IEDi at terminal i of the protected line is negative, i.e. (S_L_(i) < 0), then the IEDi assigns a value of Zi = 1 and communicates this value to the IEDj at the opposite end (j) of the protected line. Both line IEDs share each other’s Z values. If Zi = Zj = 1 then, a trip signal is sent to the CBs associated with the protected line.

#### The analysis of the line protection algorithm under different fault conditions is detailed as follows

1. Line protection algorithm operation under normal operation and external fault cases: During normal operation and external fault cases, IEDi at terminal i reads ILOCAL and receives IREMOTE from IEDj installed at terminal j. In this case, both currents are nearly the same magnitude and in the same direction. Then, IEDi and IEDj calculate the operating points SL(i) and SL(j), respectively, which are nearly equal to ≈ 1 and located in the restraining zone. Therefore, both IEDs assign Zi = Zj = 0.
2. Line protection algorithm operation under internal fault cases: In cases of internal faults, I_LOCAL_ has the opposite direction with respect to I_REMOTE_. As a result, the operating points SL(i) and SL(j) have nearly negative values and are located in the tripping zone accordingly. Hence, the relays assign Zi = Zj = 1, indicating a fault condition, and a trip command is issued to the associated CB to isolate the faulty line.
3. Line protection algorithm operation under outfeed internal faults with high impedance cases: A high impedance internal fault with outfeed is a challenging case. However, the proposed algorithm will analyze the rapid increase of I_LOCAL_ while I_REMOTE_ remains constant to feed the loads connected to the remote end of the protected line. As a result, the calculation of the operating points SL(i) and SL(j) will result in negative values, and they will be moved to the tripping zone accordingly. Consequently, both line relays assign Zi = Zj = 1, indicating a fault condition, and share the Z values, then issue a trip command to the associated CB.

#### Operating principle of the bus protection algorithm

1. Each bus’s attached IEDs communicate each other’s current readings through the IEC 61850 communication protocol via the local area network. Accordingly, each IED has the IN and OUT currents of the protected bus.
2. Each IED calculates ${S}_{B}\left(\mathrm{i}\right)$ according to Eq. (6):
  6 $$S_{B} \left( i \right) = {\raise0.7ex\hbox{${\frac{{d\Sigma \left( {I_{IN} - I_{OUT} + \varepsilon } \right)}}{dt}}$} \!\mathord{\left/ {\vphantom {{\frac{{d\Sigma \left( {I_{IN} - I_{OUT} + \varepsilon } \right)}}{dt}} {\frac{{d\Sigma \left( {I_{OUT} - I_{IN} + \varepsilon } \right)}}{dt}}}}\right.\kern-0pt} \!\lower0.7ex\hbox{${\frac{{d\Sigma \left( {I_{OUT} - I_{IN} + \varepsilon } \right)}}{dt}}$}}$$

Where SB is the calculated value for the protected bus as defined in Eq. (6), while i denotes the IED’s number, and the value of ε is a very small coefficient taken by 1% of the rated line current of any of the connected lines to the protected bus to avoid dividing by zero.

Each IED continuously monitors its operational point SB; if the IED calculated the operating point and resulted in a negative value (i.e., SB < 0), then the IED assigns a value of Z = 1 and shares the Z values with other IEDs located at the same bus. If the Z value of all buses’ IEDs is equal to 1, then a trip signal is sent to the associated CBs to disconnect the faulted bus.

#### The analysis of the bus protection algorithm under different fault conditions is analyzed as follows:

1. Bus protection algorithm operation under normal operation and line fault cases: During normal operation or line fault, the input bus current I_IN_ is almost equal to I_OUT_ output from the bus and has the same direction. Then, each IED_i_ calculates its operating point SB(i), which is nearly equal to 1 and located in the restraining zone. Hence, each bus IED assigns Z_i_ = 0.
2. Bus protection algorithm operation under internal bus fault cases: During the internal bus fault, all currents feed the fault. Hence, I_IN_ has an opposite direction to I_OUT_. Therefore, the operating point of each IED_i_ has nearly a negative value (i.e., SL(i) < 0) and is located in the tripping zone accordingly. Thus, each IED connected to the protected bus assigns Z_i_ = 1 and shares the Z values indicating a fault condition, and a trip command is issued to the associated CB.

The MATLAB script used for this algorithm is publicly available in Zenodo repository under 10.5281/zenodo.18402079

## Simulation results and evaluation

**Fig. 6:**
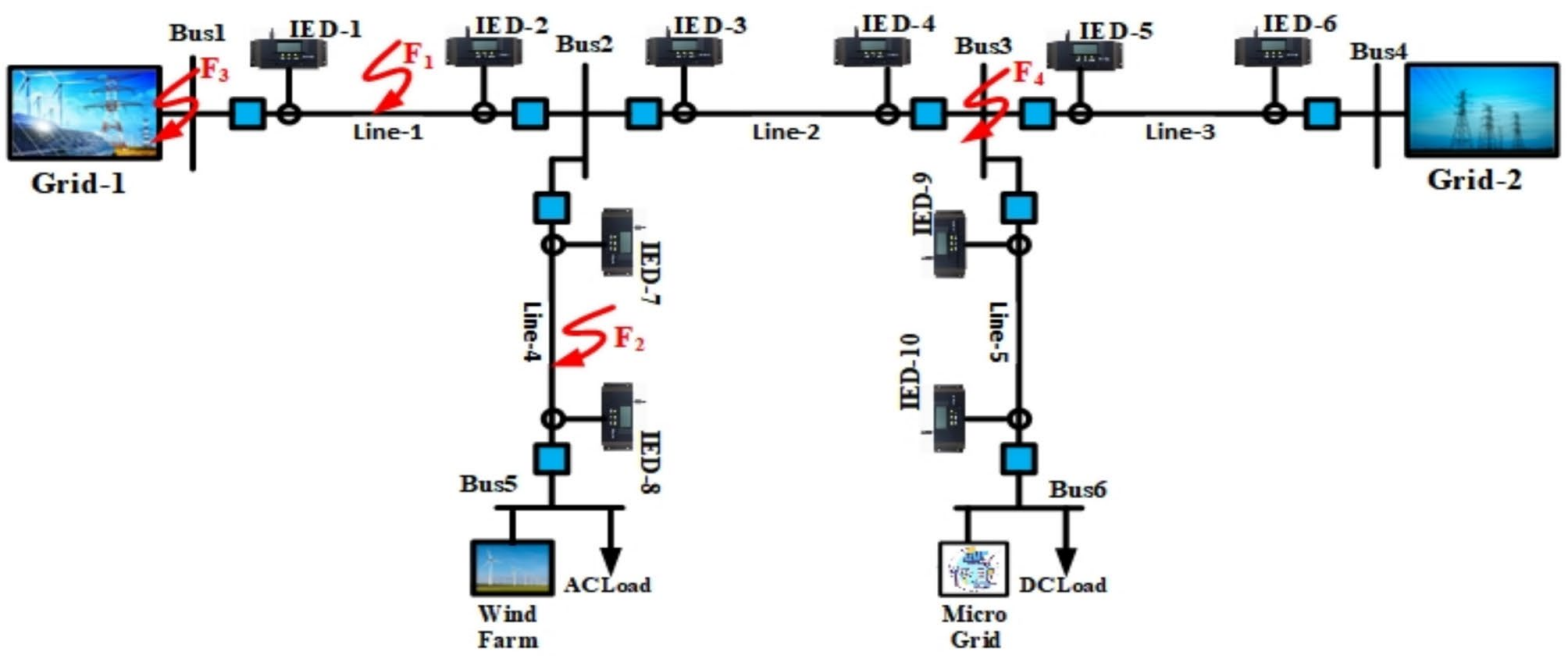
Location of the different simulated faults for the two terminal MVDC network under study.

The two-terminal 33 kV MVDC distribution network, illustrated in Fig. 1, is simulated using the PSCAD software platform to model fault current behavior. The MATLAB platform is employed for signal processing and the implementation of the trip decision algorithm. While modeling the cables, the conductor and insulation qualities, ground impedance information, and geometric position are all taken into account. Moreover, all frequency-dependent effects of cables are represented using the Frequency Dependent Phase model. A 50 kHz sampling frequency is employed for simulation. Normal and abnormal scenarios were simulated under various operating conditions, including different fault types, fault locations, fault resistances, sudden load change, DC bus fault, and AC side fault scenarios to evaluate the effectiveness of the proposed approach. The total simulation time is set at 0.25 s, where the fault occurs at t = 0.2 s. Four different fault locations (F1, F2, F3 and F4) are shown in Fig. 6.

### Pole-to-pole fault

**Fig. 7:**
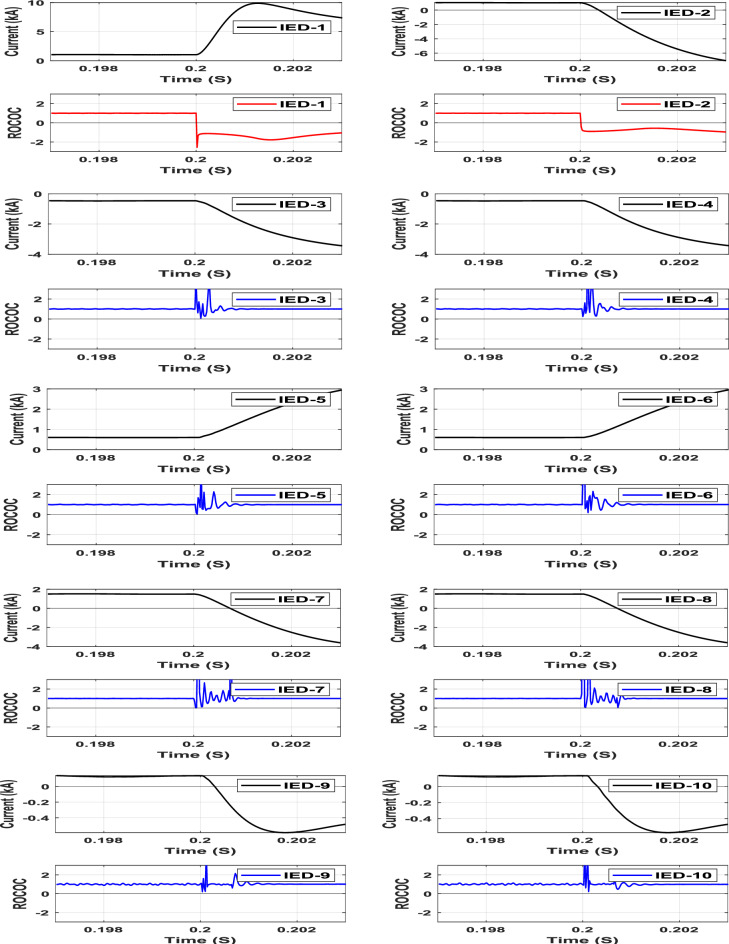
IED’s protection characteristic due to pole-to-pole fault at Line-1.

A pole-to-pole fault (F1) is simulated at the middle of Line-1. The characteristics of the entire IEDs due to the pole-to-pole fault are shown in Fig. 7. As depicted in the figure, the operating points of IEDs 1 and 2 are located in the restraining zone before the fault instant, then moved to the tripping zone following the fault instant. In contrast, the operating points of the remaining IEDs responsible for protecting other lines are located within the restraining zone during all conditions. The first operating point of the faulted line moved to the tripping zone at 0.20025 s, leading to an estimated fault detection time of 0.25 ms. Different fault locations, at the beginning, middle, and end of line-1 are simulated. The different fault locations produced the same results, indicating that the proposed scheme is not fault location-dependent and is effective regardless of DC fault location.

### Pole to ground fault

**Fig. 8:**
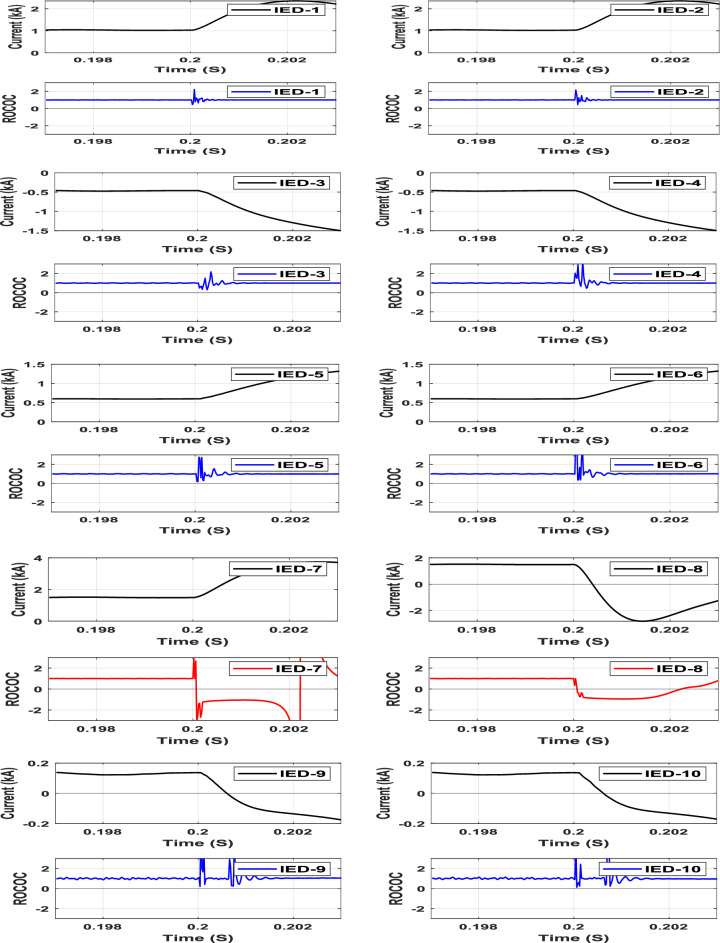
IED’s protection characteristic due to Pole-to-ground fault through 50 Ω resistance at line 4.

A pole-to-ground fault (F2) is simulated at Line-4 through different fault resistances varying from 1 to 100 ohms. The operating points of all IEDs through the characteristic due to a 50 Ω pole-to-ground fault at Line-4 are shown in Fig. 8. The operating point characteristics of IEDs 7 and 8, which are deployed at both Line-4 terminals, are the only ones moved from the restraining zone to the tripping zone among the other IEDs due to the pole to ground fault (F2) condition. The first operating point of the faulted line moved to the tripping zone at 0.20025 s for this case, implying that the proposed DC protection scheme is sensitive enough to detect high-resistance faults.

### AC side fault

**Fig. 9:**
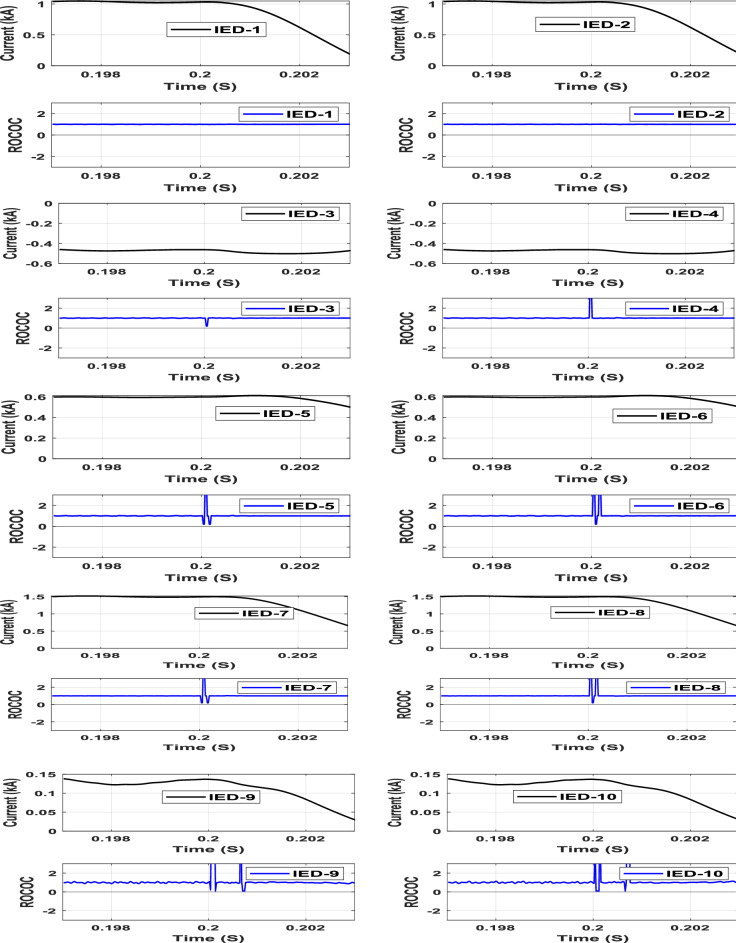
IED’s protection characteristic plan due to 3-phases fault on the AC grid.

A three-phase fault (F3) on the AC side at Grid-1 is studied in this section. The characteristics of the different IEDs due to the AC fault are displayed in Fig. 8. When the AC fault occurs, the DC voltage of the VSC (1) drops to less than 33 kV, and no power is supplied to the system. While the VSC (2) maintains the DC voltage and supplies power to the DC system. The operating points of all IEDs are observed in the restraining zone during the AC fault, as noticed in Fig. 9. As a result, the proposed DC protection scheme is therefore restrained against AC-side faults.

### Sudden load changing

**Fig. 10:**
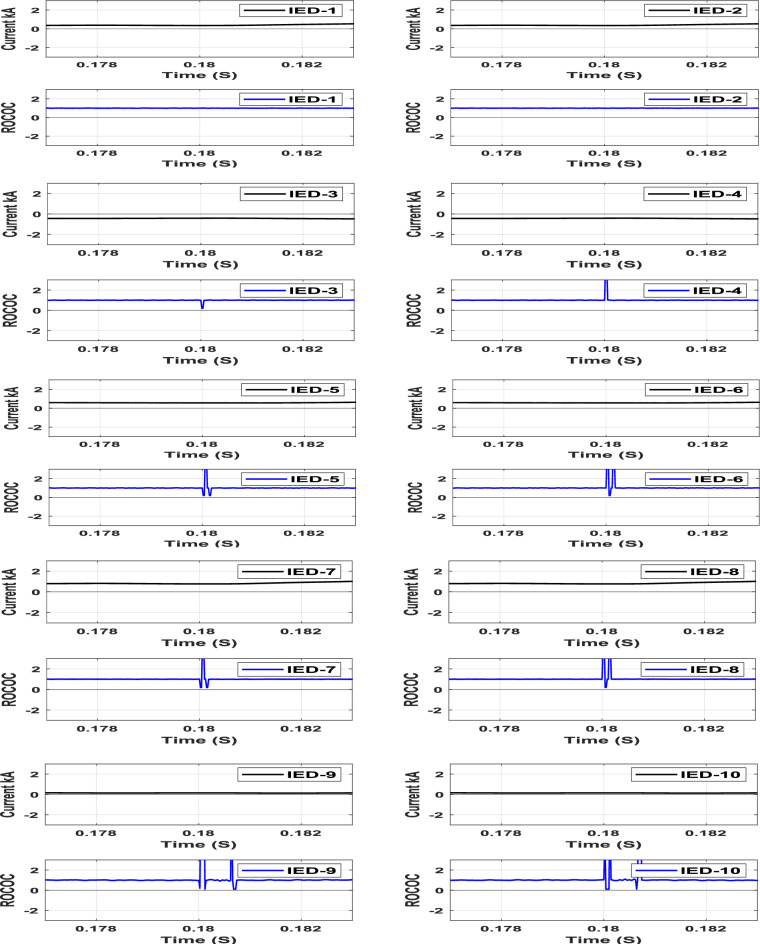
IED’s protection characteristic plan due to sudden AC load change at bus (5).

A 20 MW sudden AC load encroachment is simulated at Bus-5 at t = 0.18 s. The characteristics of the IEDs due to the sudden load change are shown in Fig. 10. The operating points of all IEDs are clearly located in the restraining zone, regardless of the sudden load change, as shown in Fig. 10. Thus, the proposed scheme restrains the sudden load change accordingly.

### Bus fault

**Fig. 11:**
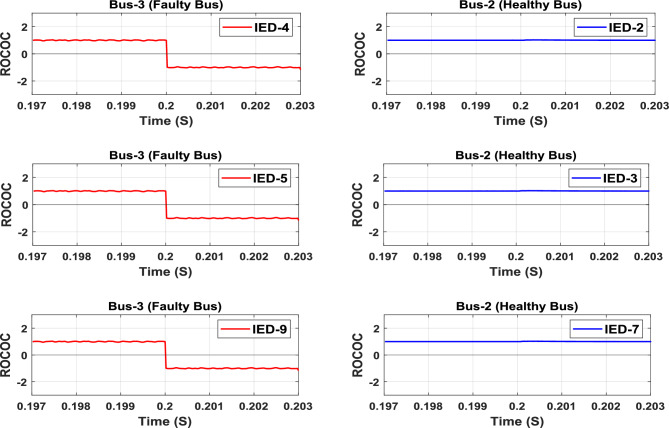
Bus’s protection characteristic plan due to fault at Bus (3).

A pole-to-pole bus fault (F4) is simulated at Bus-3. The characteristics of the group of IEDs responsible for Bus-2 and Bus-3 protection are shown in Fig. 11 as an example of healthy and faulty buses, respectively. The operating points of the IEDs (4, 5, and 9) responsible for Bus-3 protection are moved to the tripping zone after the bus fault occurrence, as noted in Fig. 11. While the operating points of the IEDs (2, 3, and 7) responsible for Bus-2 protection are still in the restraining zone. Hence, the trip signals will be issued to the associated Bus-3’s circuit breakers to isolate the faulted bus.

### Internal high-impedance fault of 200 Ω under outfeed conditions with 50 dB Gaussian noise.

**Fig. 12:**
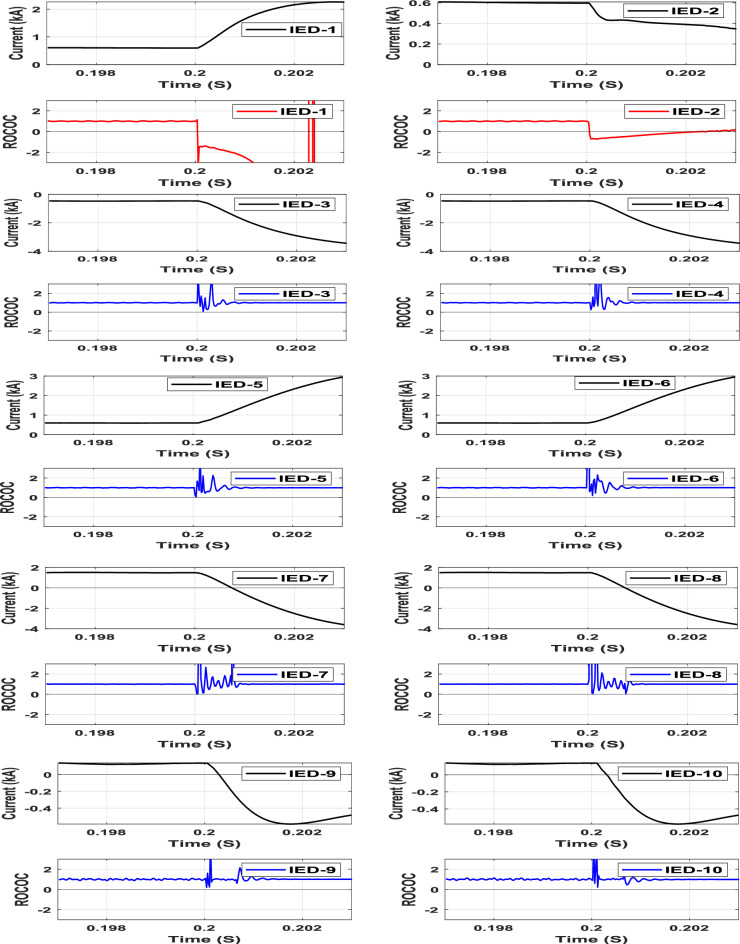
IED’s protection characteristic due to Pole-to-ground fault with outfeed through 200 Ω resistance at line-1.

To simulate the outfeed fault, the AC grid-2 is disconnected, and a pole-to-ground fault (F1) is simulated at Line-1 through a 200 Ω fault resistance with 50 dB noise. The characteristics of all system IEDs due to the outfeed fault at Line-1 are shown in Fig. 12. The operating point characteristics of IEDs 1 and 2, which are deployed at both Line-1 terminals, are the only ones moved from the restraining zone to the tripping zone among the other IEDs due to the fault (F1) condition at 0.2005s. The results of this case imply that the proposed DC protection scheme is sensitive enough to detect high-resistance faults.

### Comparative evaluation of the proposed fault detection scheme against existing protection techniques

Table 2 presents a comparative analysis of various existing fault detection methods for MVDC networks based on key performance parameters, including line discharge current (LDC) consideration, detection of internal faults with outfeed, sampling rate, fault resistance handling, noise immunity, threshold dependency, and detection time.

**Table 2:** Comparison of fault detection methods in MVDC networks.

Reference	LDC consideration	Internal fault with outfeed	Sampling rate	Fault resistance (Ω)	Noise immunity (dB)	Threshold dependency	Detection time (ms)
^16^	No	No	10 kHz	10	Not specified	Yes	2
^17^	No	No	1 MHz	10	20	Yes	-
^18^	Yes	No	10 kHz	100	20	Yes	0.5
^19^	Ignored	No	50 kHz	5	No	Yes	1—12
^20^	Yes	No	50 kHz	100	30	Yes	-
Proposed Scheme	Yes	Yes	50 kHz	200	50	No	0.5

The proposed fault detection scheme outperforms existing methods by addressing critical limitations in multiterminal MVDC networks. It effectively detects internal faults with outfeed, ensuring comprehensive fault coverage, and achieves the fastest detection time of 0.5 ms. With robust noise immunity up to 50 dB and fault resistance handling up to 200 Ω, it enhances reliability under varying conditions. Unlike conventional methods, it eliminates threshold dependency, improving sensitivity and adaptability. Overall, the scheme offers a more reliable, efficient, and robust fault detection solution for MVDC networks.

## Conclusion

Protecting MVDC systems poses inherent challenges due to the absence of natural current zero-crossings and the rapid rise of fault currents. This work introduces a novel, setting-less protection scheme that eliminates the need for predefined thresholds and enables accurate and rapid fault detection under a wide range of operating conditions.

The algorithm relies on the computed nominal value of the rate of change of differential current between local and remote terminals, enhancing protection performance while simplifying implementation. Simulation results demonstrate that the proposed method:

- Achieves fault detection times between 0.25–0.5 ms,
- Accurately detects high-resistance faults up to 200 Ω,
- Maintains robustness in the presence of up to 50 dB Gaussian noise,
- Shows immunity to sudden load changes and AC-side faults, and
- Effectively identifies internal faults with outfeed conditions.

These outcomes are validated across multiple fault types, locations, and scenarios within a two-terminal MVDC network. The scheme also incorporates advanced communication protocols (IEC 61850 and IEEE 1588) to ensure low-latency, synchronized operation between intelligent electronic devices (IEDs). In addition, the method successfully mitigates false tripping caused by line discharge currents.

Overall, the proposed setting-less protection approach demonstrates superior performance compared to traditional methods. Its threshold independence, noise resilience, sensitivity to high-impedance faults, and ultra-fast response make it a compelling candidate for practical deployment in future MVDC systems, supporting enhanced system stability, selectivity, and security.

## Supplementary Information

Supplementary Information.

## Data Availability

All data generated or analyzed during this study are included in this published article.
