# Supplementary material for: A settingless fault detection approach for MVDC network
Source: Sci Rep. 2026 Mar 4;16:8267. doi: 10.1038/s41598-026-38187-2 (PMC12963459; doi:10.1038/s41598-026-38187-2)
Supplement: Supplementary file 1 — Supplementary Information. [file 41598_2026_38187_MOESM1_ESM.docx]

The analysis code for this study has been deposited in Zenodo at <https://doi.org/10.5281/zenodo.18402079> and is also provided as a Supplementary Software file.

Matlab

% ProtectionAlgorithm.m

% Description:

% This script implements the proposed protection scheme.

% It processes the raw pole currents to extract the relevant operating mode

% (Common Mode) and calculates the Operating Points (OPL & OPR).

%% 1. Load Simulation Data

filename = 'PSCAD_Data.xlsx';

data = readtable(filename);

% Extract Time and Raw Pole Currents

Time = data.Time;

IL1SP = data.IL1SP; % Local Positive Pole Current

IL1SN = data.IL1SN; % Local Negative Pole Current

IL1RP = data.IL1RP; % Remote Positive Pole Current

IL1RN = data.IL1RN; % Remote Negative Pole Current

%% 2. Modal Transformation

% Calculate the Common Mode (Ground Mode) current component.

% This mode is derived by averaging the positive and negative pole currents.

IL = (IL1SP + IL1SN) / 2; % Calculated Local Mode Current

IR = (IL1RP + IL1RN) / 2; % Calculated Remote Mode Current

%% 3. Algorithm Calculations

% Calculate Differential Currents

Idiff_L_R = IL - IR;

Idiff_R_L = IR - IL;

% Calculate Time Step (dt)

dt = [diff(Time); 0];

dt(dt == 0) = eps; % Prevent division by zero

% Calculate Derivatives (d/dt) using numerical differentiation

d_Idiff_L_R_dt = [diff(Idiff_L_R); 0] ./ dt;

d_Idiff_R_L_dt = [diff(Idiff_R_L); 0] ./ dt;

% Algorithm Tuning Constant

k = 100;

% --- Calculate Local Operating Point (OPL) ---

% Equation: OPL = (d(Il-Ir)/dt + Il/k) / (d(Ir-Il)/dt + Ir/k)

numerator_L = d_Idiff_L_R_dt + (IL ./ k);

denominator_L = d_Idiff_R_L_dt + (IR ./ k);

OPL = numerator_L ./ denominator_L;

% --- Calculate Remote Operating Point (OPR) ---

% Equation: OPR = (d(Ir-Il)/dt + Ir/k) / (d(Il-Ir)/dt + Il/k)

numerator_R = d_Idiff_R_L_dt + (IR ./ k);

denominator_R = d_Idiff_L_R_dt + (IL ./ k);

OPR = numerator_R ./ denominator_R;

%% 4. Visualization

figure('Name', 'Protection Algorithm Results');

% Plot OPL

subplot(2,1,1);

plot(Time, OPL, 'b', 'LineWidth', 1.5);

title('Local Operating Point (OPL)');

xlabel('Time (s)');

ylabel('OPL Value');

grid on;

% Plot OPR

subplot(2,1,2);

plot(Time, OPR, 'r', 'LineWidth', 1.5);

title('Remote Operating Point (OPR)');

xlabel('Time (s)');

ylabel('OPR Value');

grid on;

%% 5. Save Processed Results

% Save the computed operating points to a generic CSV file

results = table(Time, IL, IR, OPL, OPR);

writetable(results, 'Protection_Scheme_Results.csv');

disp('Calculation complete. Results saved to Protection_Scheme_Results.csv');
